# Endothelial Delta-like 4 (DLL4) promotes renal cell carcinoma hematogenous metastasis

**DOI:** 10.18632/oncotarget.1827

**Published:** 2014-03-14

**Authors:** Qing Bo Huang, Xin Ma, Hong Zhao Li, Qing Ai, Shang Wen Liu, Yu Zhang, Yu Gao, Yang Fan, Dong Ni, Bao Jun Wang, Xu Zhang

**Affiliations:** ^1^ Department of Urology/State Key Laboratory of Kidney Diseases, Chinese PLA General Hospital/PLA Medical School, Beijing, China; ^2^ Department of Urology, Chinese PLA 303 Hospital, Nanning, China; ^3^ Department of Urology, Zhongnan Hospital of Wuhan University, Wuhan, China

**Keywords:** Kidney neoplasms, Neoplasm metastasis, Cell communication, Signal transduction, Angiogenesis

## Abstract

The Notch ligand Delta-like 4 (DLL4) plays an important role in tumor angiogenesis, which is required for tumor invasion and metastasis. Here we showed that DLL4 was elevated in endothelium and Notch signaling was activated in renal cell carcinoma (RCC). Exogenous DLL4 induced RCC cell migration and invasion by activating intercellular Notch signaling. Importantly, the DLL4/Notch/Hey1/MMP9 cascades connecting the endothelium to the cancer cells in metastasis were identified. Knockdown of Hey1 decreased expression of MMP9 and attenuated tumor invasion. The clinical investigation on 120 cases of RCC specimens indicated that expressions of Hey1 and MMP9 correlated with DLL4 density. Moreover, univariate and multivariate analyses showed that tumor hematogenous metastasis not only was depended on microvessel density but was also associated with tumor size and DLL4 density. During 4-year surveillance, high-level of DLL4 density was associated with a higher probability of developing metastasis and being sensitive to target therapies. Our data suggest that RCC progression is caused in part by activated DLL4/Notch signaling, interaction of endothelium and cells, which can be therapeutically targeted.

## INTRODUCTION

Renal cell carcinoma (RCC) is the most lethal of all urological malignancies [1], accounting for 2%–3% of adult malignancies and approximately 30% of metastatic lesions detected at initial diagnosis [2]. However, the mechanism of metastasis has not yet been fully uncovered. Moreover, the fact that RCC resists chemotherapy and radiotherapy lessen our effective systemic therapies for advanced metastatic disease.

RCC is a vascular–rich neoplasm. Thus, a better understanding of the underlying mechanisms of angiogenesis and tumor progression may help improve treatment effectiveness. Folkman et al. proposed that angiogenesis was required for invasive tumor growth and metastasis [3, 4]. This hypothesis was based on the fact that newly formed, leaky blood vessels not only promote tumor growth by providing a richly blood supply but also allow tumor cells to enter the circulation system and permit the shedding of cells from the primary tumor [5]. However, clinical observations have shown that angiogenesis was not the sole factor determining metastasis [6]. Thus, we hypothesize that blood vessels expressing angiogenesis–specific factors that are pro– or anti–tumor growth or metastasis directly communicate with tumor cells. One such vascular–specific factor is DLL4, which collaborates with vascular endothelial growth factor (VEGF) to initiate important cascades that control tumor angiogenesis and tumor progression [7, 8]. During tumor angiogenesis, DLL4 expression stimulated by VEGF is largely restricted to the tip cells of developing arteries, where it regulates the number of tip cells to control vessel sprouting and branching triggered by VEGF [7, 9].

DLL4 is a ligand of the Notch signaling pathway, which is activated by cell–cell contact between signal–sending cells that express Notch ligands and signal–receiving cells that express Notch receptors. Upon specific ligand binding, the Notch intracellular domain (NICD) is cleaved by γ–secretase, released, and then enters the nucleus and targets downstream genes that function in cell– and context–specific manners [10-12]. During angiogenesis, the sprouting blood vessels spread into the tumor cell population and they lack a complete surrounding membrane, offer an opportunity for interaction between endothelial cells and tumor cells [13]. Following these leads, we hypothesized that endothelial DLL4 may accelerate tumor progression by endothelial–tumor cell interactions.

## RESULTS

### Clinocopathologic Characteristics of RCC Samples

Demographic, clinical, and histopathologic variables are shown in Table 1. The median age was 51 years (range, 20-81 years) and the median size of tumor was 6 cm (range, 1.5-17.5 cm). To differentiate metastatic status, non-metastatic (NM) samples were obtained from primary sites without lymphatic or distant metastases; lymphatic metastatic (LM) samples were from primary sites with lymph node metastasis; hematogenous metastatic (HM) samples were from primary sites in the presence of distant metastases but absence of lymph node metastases. There were 20 patients with HM and 8 cases of LM, whereas 92 patients without metastasis. The RCC tumors comprised 90 clear cell RCC (ccRCC), 21 papillary RCC (pRCC), and 9 chromophobe RCC (chRCC).

**Table 1:** The features of the patients and the tumor tissue samples detected

Variables	No. (%)	Variables	No. (%)
Gender		Grade	
male	90 (75.0)	1	87 (72.5)
female	30 (25.0)	2	26 (21.7)
Age (y)		3	7 (5.8)
≤40	15 (12.5)	Clinical stage	
>40,≤60	75 (62.5)	I	54 (45.0)
>60	30 (25.0)	II	14 (11.7)
BMI		III	24 (20.0)
≥ 18.5	5 (4.2)	IV	28 (23.3)
≥18.5, <24	44 (36.7)	T stage	
≥24, <28	51 (42.5)	T1	61 (50.8)
≥28	20 (16.7)	T2	21 (17.5)
Classification		T3	32 (26.7)
ccRCC	90 (75.0)	T4	6 (5.0)
pRCC	21 (17.5)	Metastatic status	
chRCC	9 (7.5)	NM	92 (76.7)
Tumor size (cm)		LM	8 (6.7)
≤4	35 (29.2)	HM	20 (16.7)
>4, ≤7	36 (30.0)	Necrosis	
>7, ≤10	29 (24.2)	no	61 (50.8)
>10	20 (16.7)	yes	59 (49.2)

BMI: body mass index, reference to Chinese standard; ccRCC: clear cell renal cell carcinoma; pRCC: papillary renal cell carcinoma; chRCC: chromophobe renal cell carcinoma; NM: tumors involving non-metastasis; LM: tumors involving lymphatic metastases; HM: tumors involving hematogenous metastases.

### Clinical Association of Angiogenesis–specific DLL4 with Hematogenous Metastasis of RCC

**Figure 1:**
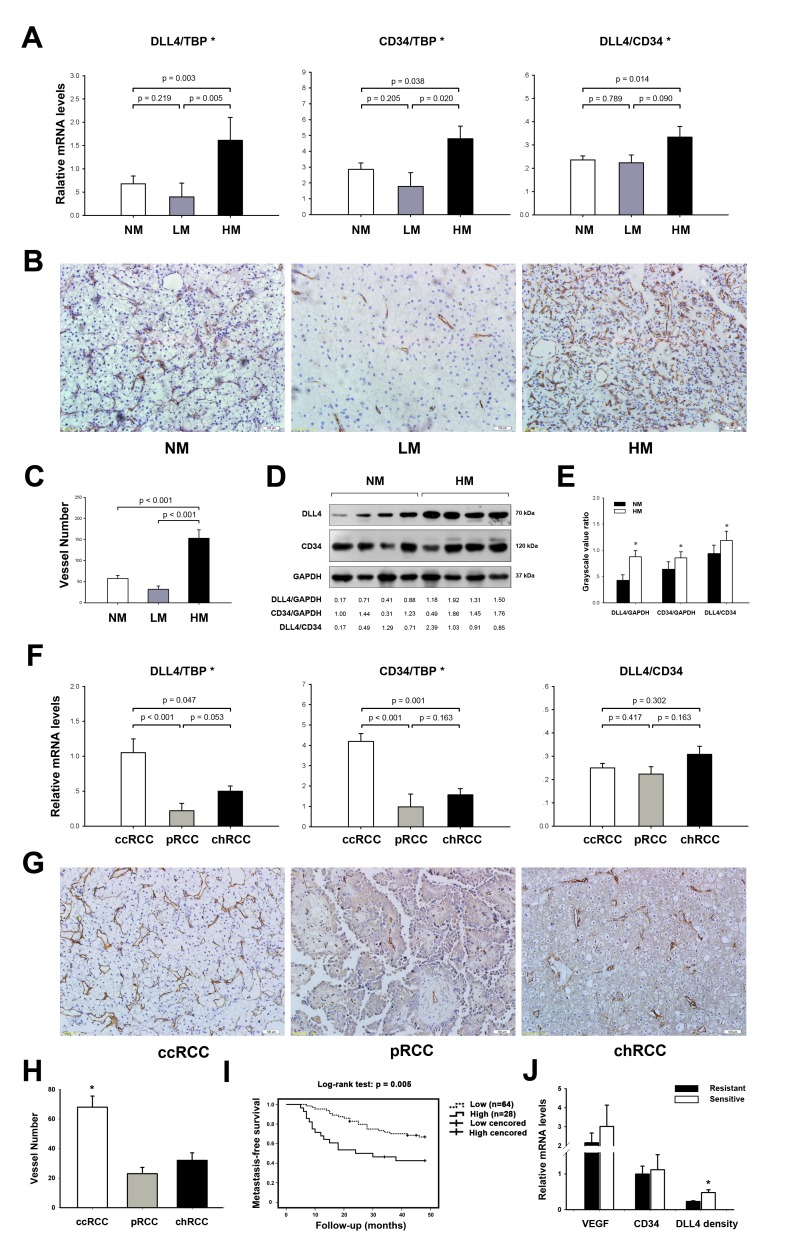
Clinical association of DLL4 with the hematogenous metastasis of RCC (A) real–time PCR analyses of total DLL4 expression (DLL4/TBP), MVD (CD34/TBP), and DLL4 density (DLL4/CD34) in non–metastatic (NM; n = 92), lymphatic metastatic (LM; n = 8) and hematogenous metastatic RCC (HM; n = 20). *p < 0.05, statistically significant changes (between groups). (B) CD34 staining MVD in clear cell RCC (ccRCC), including NM, LM, HM. (C) Statistical results of CD34 staining MVD in HM (n = 10), LM (n = 7) and NM (n = 16) within ccRCC. (D and E) Western blot analysis of total DLL4, CD34, and DLL4 density in HM (n = 12) relative to NM (n = 12). The numbers shown below are the grayscale ratio of the corresponding proteins on the left and analyzed in (E). (F) real–time PCR analyses of total DLL4 expression, MVD and DLL4 density in ccRCC (n = 90) compared with pRCC (n = 21) and chRCC (n = 9). (G) CD34 staining MVD in ccRCC, pRCC and chRCC. (I) Kaplan–Meier graph representing the probability of metastasis–free survival in RCC without synchronous metastases stratified by low–level of high–level of DLL4 density. The log–rank test p value reflects the significance of the association between DLL4 density and metastasis. (J) real–time PCR analyses of mRNA expressions of VEGF, CD34 and DLL4 density in tumors grouped by sensitive or resistant to target therapies. Data represent the means ± SEM. * statistics significant.

The expressions of DLL4/Notch signaling components in RCC tissue samples were detected and shown in Figure S2. DLL4, Notch1, Notch2 and downstream targets Hey1 and Hey2 were up–regulated in RCC tissues and DLL4 was validated to localized on endothelium previous [14]. A multivariate analysis method called logistic regression model was constructed to selected factors associated with RCC hematogenous metastasis. Tumor metastasis status (hematogenous metastasis or not) was selected as dependent variable. Covariables including patient characteristics (gender, age and body mass index (BMI)), tumor features (including tumor size, histological classification, grade, and T stage), and angiogenesis associated–factors (including MVD and DLL4 density) were all transformed into binary data. The results revealed that the tumor size, MVD, and DLL4 density were correlated with hematogenous metastasis (Table 2). Specially, the risk of hematogenous metastasis in tumor expressed high-level of DLL4 density was 23.4 fold of that in tumor expressed low-level of DLL4 density. In univariate analysis, the mean tumor size in LM and HM groups (10 cm and 8.7 cm, respectively) were significant bigger than that in NM group (6.1 cm). Additionally, DLL4 expression and MVD increased from the non–metastatic (NM) and LM groups to the HM group (Figure 1A). The DLL4 density in HM was also higher than in the LM and NM groups, despite the former comparison showing only marginal significance because of small sample volumes. However, DLL4 expression, MVD, and DLL4 density were not significantly different between the LM and NM groups. Further investigation showed that CD34–staining MVD was also increased from LM and NM to HM (Figures 1B and 1C). In the Western blotting analysis, CD34 expression and DLL4 density were also significantly elevated in HM compared with NM (Figures 1D and 1E).

**Table 2:** Logistic regression analyses of hematogenous metastasis-associated factors

Valuables	HM (n, %)	B	SE	p	OR (95% CI)
Gender		−1.206	0.801	0.132	0.299 (0.062-1.439)
Male (90)	17 (18.9)				
Female (30)	3 (10.0)				
Age		0.899	0.620	0.147	2.456 (0.728-8.283)
≤60 (90)	12 (13.3)				
>60 (30)	8 (26.7)				
BMI		−0.689	0.622	0.269	0.502 (0.148-1.701)
<25 (65)	13 (20.0)				
≥25 (55)	7 (12.7)				
Classification		−0.350	1.256	0.780	0.704 (0.060-8.261)
ccRCC (90)	19 (21.1)				
pRCC&chRCC (30)	1 (3.3)				
Tumor size		1.966	0.760	0.010*	7.142 (1.609-31.702)
≤7 (71)	7 (9.9)				
>7 (49)	13 (26.5)				
Grade		0.724	0.682	0.288	2.063 (0.542-7.851)
1 (87)	11 (12.6)				
2&3 (33)	9 (27.3)				
pT Stage		−0.096	0.799	0.905	0.909 (0.190-4.352)
T1& T2 (82)	12 (14.6)				
T3& T4 (38)	8 (21.1)				
MVD		2.506	1.131	0.027*	12.254 (1.334-112.548)
Low (100)	14 (14.0)				
High (20)	6 (30.0)				
DLL4 dengsity		3.153	1.346	0.019*	23.409 (1.675-327.113)
Low (100)	14 (14.0)				
High (20)	6 (30.0)				
Constant		−3.501	3.012	0.245	0.030

Low– or high– level of DLL4 density and CD34 expression divided at the thresholds where obvious separations appear. Abbreviation: BMI: body mass index; HM: Hematogenous metastasis; OR: odds ratio; CI: confidence interval.

* statistics significant.

To our knowledge, clear cell RCC (ccRCC) is the most prevalent subtype and has the highest potential to metastasize. However, tumor classification was not an independent predictor of RCC hematogenous spread in a multivariate analysis. In univariate analysis, of interest, DLL4 expression in the ccRCC subtype was upregulated by increased MVD rather than increased DLL4 density relative to papillary RCC (pRCC) and chromophobe RCC (chRCC) (Figure 1F). CD34–staining MVD in pRCC and chRCC was also less than in ccRCC (Figure 1G and 1H). Thus, it seems that tumor subtype was not an independent factor when it was controlled by MVD in multivariate analysis model.

Because we lack imaging of a sufficient sensitivity to detect single cells, intravasation of the circulatory system and an occult micrometastasis may have occurred in the non–metastatic group when the primary tumors were resected. Thus, the patients in this cohort were prospectively followed up for a 4–year observational period. 92 patients without synchronous metastases were divided into two groups based on relatively high– or low–levels of DLL4 density. The strategy selecting threshold was described before [14]. During surveillance, 16 out of 28 cases in the high–level group developed distant metastasis, while 22 out of 64 cases occurred distant metastases in low–level DLL4 density group. Remarkably, when tested using Kaplan–Meier survival analysis, the high–level DLL4 density group displayed a significantly higher probability of developing metastasis than the low–level group (Figure 1I).

VEGF was an important upstream factor of DLL4 during tumor developing angiogenesis and metastasis [8, 15, 16], and the anti-angiogenesis therapies for metastatic RCC majorly targeted VEGF. Thus, we also determined the role of VEGF in RCC metastasis. In this study, VEGF mRNA expression in HM group was 5.6 fold of that in NM group. Moreover, DLL4 expression also positively correlates with VEGF expression (Figure S3). During 4–year follow–up, a cohort of 19 patients with metastatic clear cell RCC were administrated for at least 3 courses target therapies (Sunitinib or Sorafenib). During surveillance, 8 patients were sensitive to target therapies and 11 patients were refractory. To further characterize what conditions tumor were sensitive and resistant to anti-angiogenesis therapies, we compared the expressions of VEGF, CD34, and DLL4 density in the two groups. The results indicated that the DLL4 density in sensitive group was about 2 fold of that in resistant group. However, expressions of VEGF and CD34 showed no significant difference between the two groups (Figure 1J).

### DLL4 Promoted RCC Cell Migration and Invasion by Stimulating Metalloprotease Secretion

**Figure 2:**
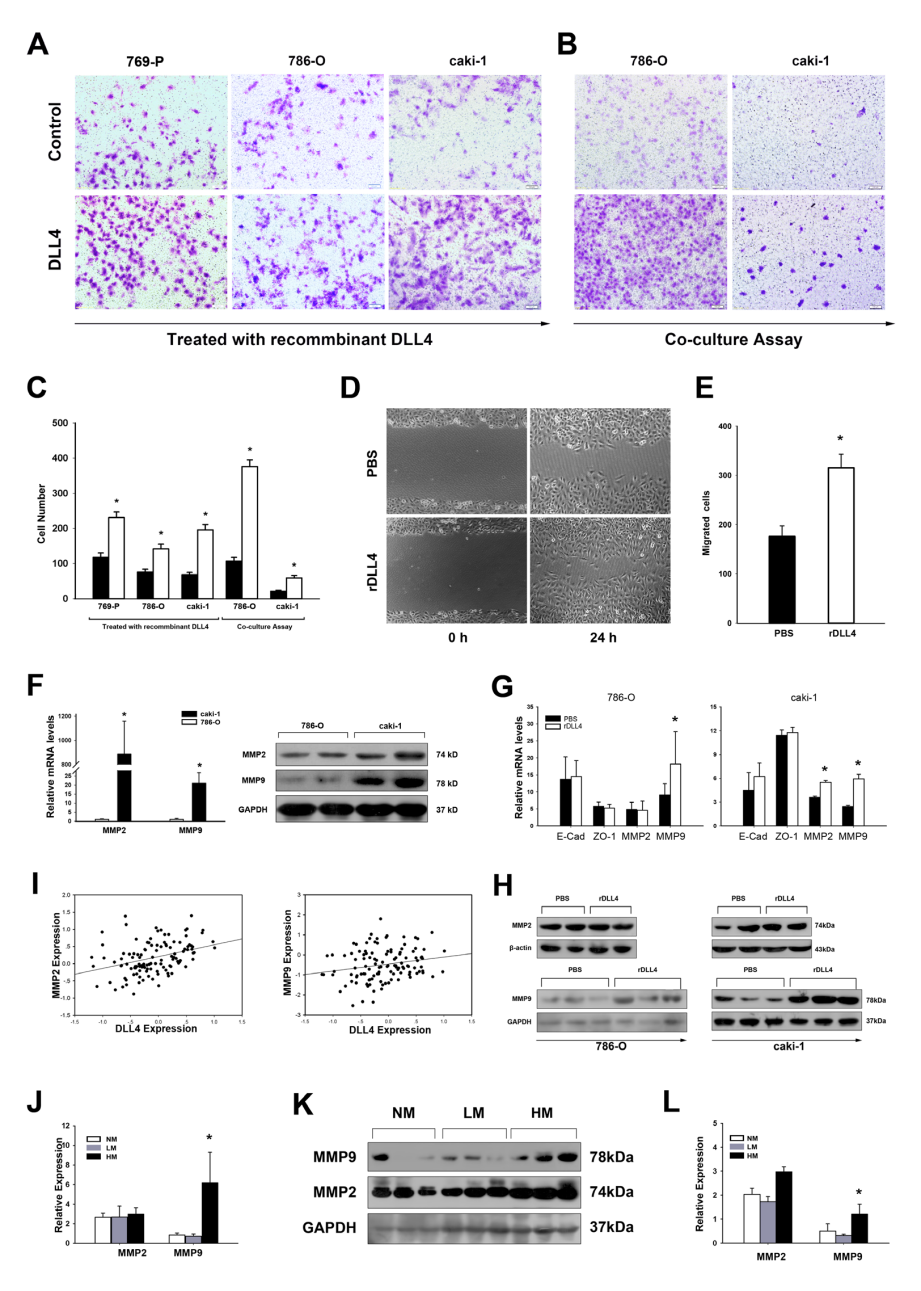
Endothelial DLL4 promotes RCC cell migration and invasion by stimulating MMP secretion (A–C) Transwell assay of RCC cells treated with recombinant DLL4 (A) or co–cultured with K562 cells expressing DLL4 (B). Data in three independent experiments were analyzed in (C). (D and E) 786–O cells treated with recombinant DLL4 (rDLL4) and negative control for 48 hours were tested in the wound healing assay at the indicated time points. The data shown are representative images. Three independent experiments were performed, and the number of migrated cells was compared in (E). (F) real–time PCR and Western blotting analyses of MMP2 and MMP9 in the metastatic cell line caki–1 relative to the non–metastatic cell line 786–O. Experiments were performed in triplicate; (G) real–time PCR analyses of 786–O and caki–1 cells by rDLL4 treatment analyzed for mRNA expressions of E-cadherin, ZO-1, MMP2 and MMP9. (H) Protein expressions of MMP2 and MMP9 were further detected by Western blotting. (I) Total DLL4 expression positively correlates with MMP2 (r = 0.381, p < 0.001, and n = 120) and MMP9 (r = 0.233, p = 0.011, and n = 120); (J and K and L) real–time PCR and Western blot analyses of MMP2 and MMP9 expression in NM, LM and HM. The grayscale values in the western blot (K) are shown in (L); Data represent the means ± SEM. * statistics significant

We then sought to determine whether DLL4 enhances the cell motility of RCC through cell–cell communication. The RCC cell lines 786–O, 769-P, and caki–1 (all cell lines express Notch1, Notch2, Figure S2) were treated with recombinant DLL4; 786–O and caki–1 were further analyzed using a co–culture assay. The capacity of invasion of these cells was increased by DLL4 stimulation according to transwell assays (Figures 2A–2C). The wound–healing assay showed that DLL4 promoted migration of 786-O cells (Figures 2D and 2E).

We next attempted to explore the underlying mechanisms. First, the expression of the cell adhesion and tight junction markers E–cadherin and ZO–1 were compared between the non–metastatic cell line 786–O and metastatic cell line caki–1, but no significant differences were found (Figure S4). The mRNA levels of E–cadherin and ZO–1 were also unchanged by the DLL4 treatments (Figure 2G). Subsequently, DLL4 induction of metastasis–associated matrix metalloproteinases (MMPs) secretion was investigated. Both MMP2 and MMP9 were upregulated in the metastatic cell line caki–1 compared with 786–O (Figure 2F). Once stimulated by DLL4, MMP2 and MMP9 expression were both elevated in caki–1, but only MMP9 was upregulated in 786–O (Figure 2G and 2H). Furthermore, both MMP2 and MMP9 positively correlated with DLL4 expression In the RCC (Figure 2I). When patients were grouped according to metastatic status, MMP9 was also upregulated in the HM group (Figures 2J to 2L). The mRNA level of MMP9 in HM was 7.36–fold higher than that in NM and 8.71–fold higher than that in LM, despite the latter showing no statistical significance.

### Elevation of Hey1 in RCC Cells Mediated the Metastatic Function of Endothelial DLL4

**Figure 3:**
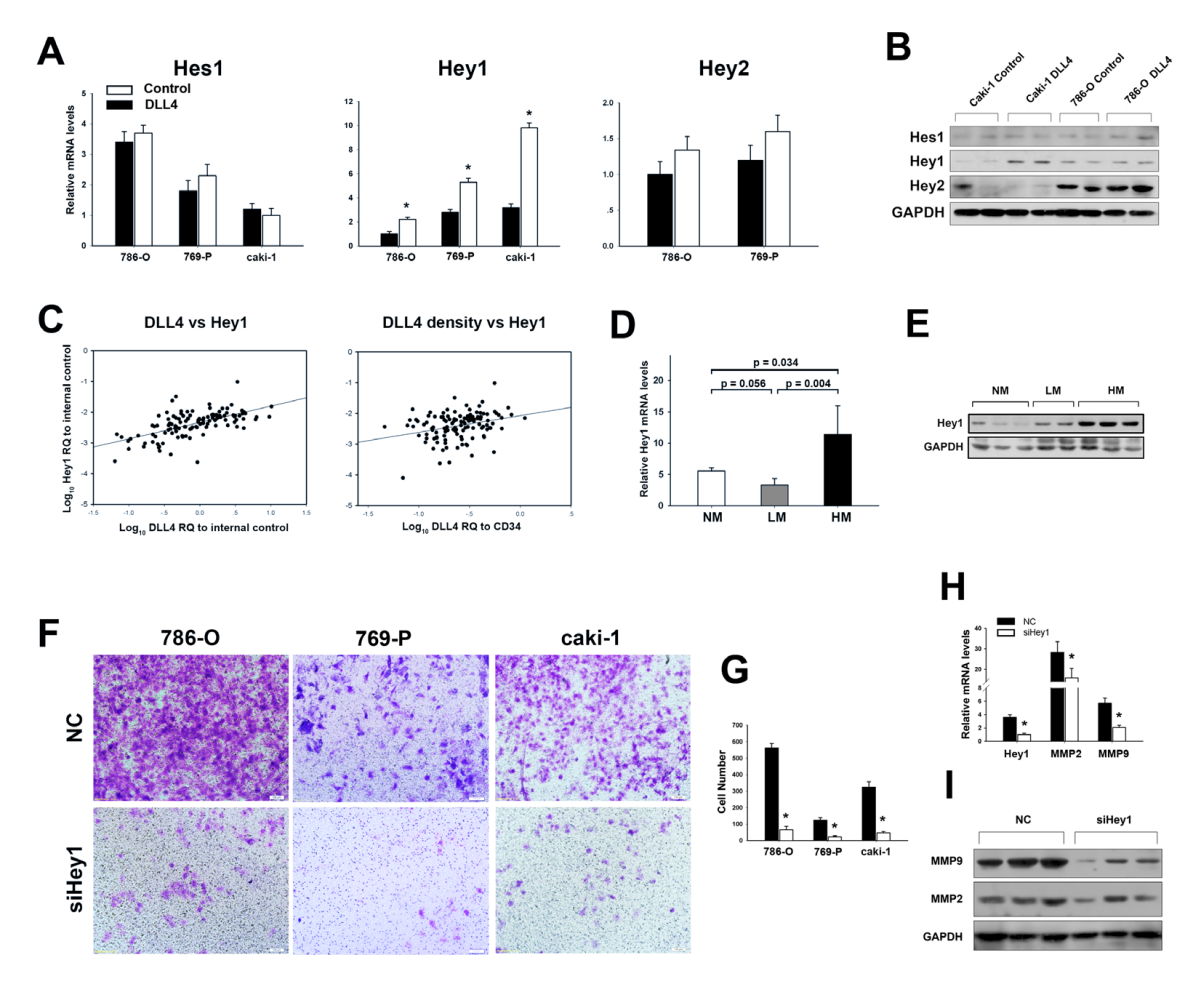
Hey1 mediates DLL4/Notch signaling in RCC hematogenous metastasis (A) Hes1, Hey1 and Hey2 mRNA levels assessed by real–time PCR in RCC cells 48 h after recombinant DLL4 or control treatment. The data shown are from three independent experiments. (B) Western blot analyses of 786–O and caki–1 cells treated with recombinant DLL4 or control for 72 hours. (C) Hey1 expression positively correlates with both total DLL4 expression (r = 0.633, p < 0.001, and n = 120) and DLL4 density (r = 0.303, p = 0.01, and n = 120). (D) mRNA levels of Hey1 in HM relative to LM or NM. (E) Representative Western blot of Hey1 expression in HM relative to LM or NM. (F and G) Invasiveness of RCC cells with Hey1 knockdown or control monitored by transwell assay. Data in three independent experiments were analyzed in (G). (H) Knockdown of Hey1 decreases MMP2 and MMP9 mRNA levels. (I) Representative experiment showing that Hey1 knockdown decreases MMP2 and MMP9 protein levels. Data represent the means ± SEM. * statistics significant.

We next sought to identify key downstream mediators of Notch signaling in RCC cells once accepting adjacent intercellular DLL4 signaling. The Hes and Hey family members Hes1, and Hey2 were not significantly changed by DLL4 stimulation, but Hey1 was upregulated in all cells after DLL4 treatment (Figures 3A and 3B).

To confirm that Hey1 was the major downstream factor of DLL4/Notch signaling during RCC metastasis, expressions of Hes1, Hey1 and Hey2 were detected in the RCC samples using real–time PCR. We found that DLL4 expression was moderately correlated with Hey1 (Figure 3C) but poorly correlated with Hes1 (Figure S5A) and Hey2 (Figure S5B). Notably, Hey1 also increased with DLL4 density, which excluded the influence of MVD (Figure 3C). However, no correlations were observed between DLL4 density and Hes1 or Hey2 (Figure S5C and S5D). In addition, Hey1 expression was upregulated in HM (Figures 3D and 3E), but no significant differences in Hes1 and Hey2 expressions were observed between the three groups (Figure S5E and S5F). Thus, the current study focused on whether Hey1 mediated the metastatic effect of DLL4. As shown in Figures 3F and 3G, the metastatic capacities were strikingly reduced by down–regulation of endogenous Hey1 in both the non–metastatic RCC cell lines 786–O and 769-P and the metastatic cell line caki–1. Knockdown of Hey1 in caki-1 cells also decreased MMP2 and MMP9 (Figures 3H and 3I), which were upregulated by DLL4 stimulation.

## DISCUSSION

Renal cell carcinoma (RCC) is a highly vascularized tumor with frequent hematogenous metastasis, especially in large and advanced–stage tumors. Small renal masses and localized RCC can be approached with nephron–sparing surgery or radical nephrectomy and are associated with favorable survival. However, we lack effective systematic therapies for metastatic advanced–stage RCC, in which the first line pharmacotherapy is anti–angiogenic. Thus, the mechanisms underlying how angiogenesis promotes hematogenous metastasis should be investigated.

DLL4 signaling has been extensively reported to be critical for tumor angiogenesis. Blockage of DLL4 signaling inhibits tumor growth by deregulating angiogenesis and promoting non–productive angiogenesis [17, 18]. Additionally, DLL4 has been shown to help regulate the cellular actions of VEGF. Mechanistically, tumor–derived VEGF induces DLL4 expression in sprouting endothelial cells (tip cells), which then provide signals to adjacent downstream Notch receptor–bearing endothelial cells (stalk cells) to down–regulate VEGF–induced sprouting and branching [9, 19]. Under control of these two signaling pathways, angiogenesis maintain balance with tumor growth.

Interaction of endothelial cells and cancer cells were reported to promote tumor progression by generating new vessels or an invasive phenotype of cancer cells [20, 21]. A few investigations focused on the effect of DLL4 in tumor progression via cell–cell communication. Indraccolo. et al reported endothelial DLL4-Tumor-Notch interactions made tumor overcome dormancy [22]. Ding et al. reported that DLL4/Notch mediated cross–talk between endothelial cells and tumors, which suppressed lung cancer growth [13]. In our previous investigation, a DLL4 regulated microRNA named miR-30a was down-regulated in hematogenous metastatic ccRCC [14]. Under this or other unclear specific circumstances, DLL4 expression density increased in cases of RCC with hematogenous metastasis, which indicated that the up–regulation of DLL4 may enhance the metastatic capability of RCC cells. Multivariate logistic analysis of RCC specimens showed that tumor hematogenous metastasis not only depended on angiogenesis but was also associated with tumor size and DLL4 density. During hematogenous metastasis, tumor cells must invade the tissue surrounding the primary tumor, enter the bloodstream, survive and eventually arrest in the circulation, extravasate into a tissue and grow at the new site [23]. Invasion into the bloodstream is the first step for RCC metastasis, considering that localized RCC, especially nonmetastatic small RCC, demonstrates low rates of metastasis [24]. Folkman et al. proposed that tumor metastasis might depend on angiogenesis, which allows the cells access to blood vessels in which to travel [3, 4]. In addition to angiogenesis, accumulating evidence suggests that tumor metastasis is associated with tumor size [24-26]. As tumor size increases, more tumor angiogenesis occurs, leading to increasing numbers of microvessels, for more which may allow more cells to enter the blood stream. Finally, the role of up-regulated DLL4 density in RCC metastasis should not be ignored. During a 4–year surveillance period, a high level of DLL4 density was associated with higher rates of metastasis. Functionally, the migration and invasion capacities of RCC cells were directly enhanced by DLL4–Notch binding. Increased MVD may contribute to hematogenous metastasis by either offering a mode of transport through blood vessels or increasing total DLL4 levels.

Disruption of the basement membrane allows cancer cells into blood vessels, which initiates hematogenous metastasis. To our knowledge, the proteolytic activity of MMPs affects many components of the basement membrane and extracellular matrix [27]. MMP2 and MMP9 have been particularly associated with tumor progression, metastatic dissemination, and poor survival in different human cancers, including ccRCC [27-30]. In the present study, angiogenesis, with DLL4 at the leading edge of migrating endothelial cells, was proposed to aid in the degradation of the extracellular matrix and facilitate RCC cell invasion by elevating MMP2 and MMP9. More importantly, the MMP secretion was Notch dependent because inhibition of the Notch effector Hey1 decreased both MMP2 and MMP9, which eventually decreased cancer cell invasion.

It was known that VEGF stimulated DLL4 expression in endothelial cells [8, 15, 16]. Lobov et al. used oxygen-induced ischemic retinopathy (OIR) model demonstrated that VEGF blockade markedly inhibited Dll4 expression at the leading front of the growing superficial vascular plexus but had no appreciable effect on Dll4 expression in differentiated arteries [16]. Interestingly, DLL4-Notch signaling was reported to mediate tumor resistance to anti-VEGF therapy [31]. Taken together, these suggested that there were other mechanisms regulated DLL4, such as post-transcriptionally regulated by miR-30a [14]. DLL4–targeted agents have recently been clinically applied as an alternative drug that targets angiogenesis [32, 33]. In the current study, we propose for the first time that endothelial DLL4 initiates RCC hematogenous dissemination through cell–cell interactions. Moreover, DLL4 density seemed to be a predictor of effectiveness of target therapy, because tumors with high–level of DLL4 density were sensitive to anti-angiogenesis drugs, while tumors with low–level of DLL4 density were anti–angiogenesis therapy refractory. Actually, attenuation of DLL4–mediated Notch signaling pathway results in a growth inhibition of both VEGF–dependent and VEGF– independent tumors in preclinical models [17, 18, 34]. Taken together, DLL4/Notch signaling, which is interconnected with VEGF signaling, is a crucial mediator of endothelium–cancer cell communication in various processes including angiogenesis and tumor metastasis.

In summary, DLL4/Notch/Hey1/MMP9 cascade mediates a direct interplay between endothelial cells and tumor cells, which eventually promotes RCC hematogenous metastasis. Approaches for disrupting this cascade may help attenuate tumor progression.

## MATERIALS AND METHODS

### Cells and tissue samples

Human RCC cell lines (769-P, 786–O, and caki–1) were obtained from and authenticated by Cell Resource Center in China. These cells were maintained in Dulbecco's modified Eagle's medium (DMEM)/F12 (HyClone, Inc., USA) supplemented with 10% fetal bovine serum (Gibco BRL, Grand Island, NY, USA).

A total of 120 cases of RCC samples and adjacent non–tumor tissues were obtained postoperatively from the Department of Urology, PLA General Hospital. All patients provided signed Informed Consent for the use of their tissues for scientific research. The current study was approved by the Institutional Review Board. The areas tumors were identified by two separate senior pathologists and staged based on the 2011 Union for International Cancer Control (UICC) TNM classification of malignant tumors.

### Follow-up data collection and definition

Approved by the institutional Review Board, we reviewed prospectively maintained, computerized medical record database from State Key Laboratory of Kidney Diseases, Chinese PLA General Hospital. After operation, every patient was followed up for 4–year period during January 2009 to May 2013. Metastasis was defined as radiological or biopsied confirmation of the same malignancy out of the renal bed. Patients who suffered metastatic clear cell RCC and received target therapies (Sunitinib or Sorafenib) were evaluated by CT or MRI according to RECIST criteria [35] once every cycle during the first four cycles, and then once every other cycle thereafter. Patients achieved partial responses or demonstrated stable disease were considered to be sensitive to target therapies, while patients developed progressive disease were defined as resistance.

### Transient transfection and drug treatments

The full–length human DLL4 (SC113239) and its corresponding empty vector PCMV6–XL6 were purchased from the OriGene Company (USA). Transient transfection was performed using the Lipofectamine 2000 reagent (Invitrogen, USA) according to the manufacturer's instructions. For siRNA transfection (Sequences see Table S1), the quantity per 6–well plate was 100 pmol siRNA and 5 μL of reagent. The transfection efficiency was evaluated by Western blot analysis (Figure S1). Recombinant human DLL4 was purchased from R&D Systems (USA), dissolved in phosphate buffered saline (PBS) and coated overnight onto tissue culture dishes at 1 μg/mL in 0.2% gelatin.

### Co–culturing assays

One day prior to co–culturing, 769-P, 786–O, and caki–1 adherent cells (2 × 10^5^) were transferred into 6–well plates. K562 non–adherent cells were transfected with the full–length DLL4–expressing vector and its corresponding empty vector. After 24 h, the K562 cells expressing high– or low–levels of DLL4 were harvested, washed three times with fresh medium, and overlaid in a suspension (5 × 10^5^) onto the adherent cells. The co–cultures were incubated for 48 h. The culture medium and unattached cells were removed from each plate, and the remaining cells were washed three times with PBS to remove tightly bound K562 cells and harvest the layer of adherent cells underneath. Cell proliferation and transwell analyses were conducted with these adherent cells.

### Real–time PCR, Western blotting and Immunohistochemical staining for MVD

These methods have been described previously [14, 36] and the primers and antibodies used are reported in Table S2 and S3, respectively.

### Transwell assays and wound healing assay

Transwell assays have been described previously [36]. For wound healing assay, the cells were scratched after treatments with recombinant DLL4 or corresponding solution. The cells migrated into the wounds were counted after 24 h. Three independent experiments were performed.

### Statistical analysis

The relative quantitation of gene expression detected by real-time PCR was log10 transformed and analyzed by student t test or ANOVA. Other statistical analysis methods were specified when used. p values less than 0.05 were considered significant.

## SUPPLEMENTARY TABLES AND FIGURES


