# Supplementary material for: Endothelial Delta-like 4 (DLL4) promotes renal cell carcinoma hematogenous metastasis
Source: Oncotarget. 2014 Mar 14;5(10):3066–75. doi: 10.18632/oncotarget.1827 (PMC4102792; doi:10.18632/oncotarget.1827)
Supplement: Supplementary file 1 [file oncotarget-05-3066-s001.pdf]

Endothelial Delta-like 4 (DLL4) Promotes Renal Cell Carcinoma Hematogenous Metastasis – Huang et al

Supplemental Material

1. Materials and Methods

Table S1. siRNA sequence

siRNA	Sense (5′–3′)	Anti–sense (5′–3′)
siHey1	GGAGAGGAAUAAUUGAGAATT	UUCUCAAUUAUUCCUCUCCTT
NC	UUCUCCGAACGUGUCACGUTT	ACGUGACACGUUCGGAGAATT

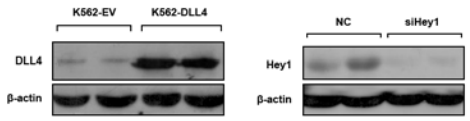

Figure S1. The transfection efficiency was evaluated by Western blot analysis before the every experiment concluding transfection. As shown above, the transfection efficiency was acceptable either in overexpressing DLL4 to K562 or knockdown of Hey1 in RCC cells.

Table S2. qRT-PCR primers

Target Genes	Sense	Antisense
DLL4	CTGCGGTTACACAGTGAA	ACATTCATTCCTCTCCTCTG
Notch1	GTGACTGCTCCCTCAACTTCAAT	CTGTCACAGTGGCCGTCACT
Notch2	CAGTTAATAATTGGTCCAT	ATTCACCTGTTAATATCTAC
Hes1	ACGTGCGAGGGCGTTAATAC	ATTGATCTGGGTCATGCAGTTG
Hey1	TTTGAGAAGCAGGGATCTGCTAAGC	CTTTCCCTCCTGCCGTATGCAGC
Hey2	TTCTCCAACAACAACATCTC	GGCACAAGTCTTCTCAAC
Ki67	TCTGTTATTGATGAGCCTGTA	GTTGACTTCCTTCCATTCTG
VEGF	AGGAGGAGGGCAGAATCATCA	CTCGATTGGATGGCAGTAGCT
CD34	TCTCCCACTAAACCCTATACA	CTTCTCTGATGCCTGAACAT
MMP2	GCGGCGGTCACAGCTACTT	CACGCTCTTCAGACTTTGGTTCT
MMP9	CCTGGAGACCTGAGAACCAATC	CCACCCGAGTGTAACCATAGC
E-cadherin	TGAGTGTCCCCCGGTATCTTC	CAGTATCAGCCGCTTTCAGATTTT
ZO-1	AAGTCACACTGGTGAAATCC	CTCTTGCTGCCAAACTATCT
TBP	CACGAACCACGGCACTGATT	TTTTCTTGCTGCCAGTCTGGAC
PPIA	TCATCTGCACTGCCAAGACTG	CATGCCTTCTTTCACTTTGCC

Table S3. Antibodies used

Antibody	Source	Company (catalog)
DLL4	Mouse monoclonal antibody	Santa Cruz (sc–365429)
Notch1	Rabbit polyclonal antibody	ABcam(ab8925)
Notch2	Rabbit polyclonal antibody	ABcam(ab8926)
Hes1	Rabbit polyclonal antibody	Santa Cruz (sc–25392)
Hey1	Goat polyclonal antibody	Santa Cruz (sc–30947)
Hey2	Rabbit polyclonal antibody	Santa Cruz (sc–28747)
CD34	Rabbit monoclonal antibody	Epitomics (2150-1)
MMP2	Rabbit	Bioworld (L638)
MMP9	Rabbit	Bioworld (W680)

2. Results

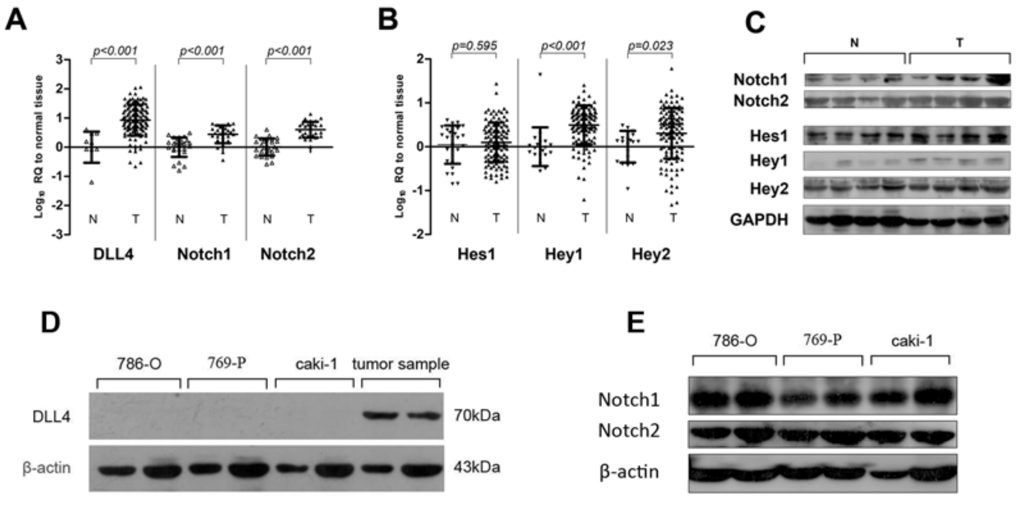

Figure S2. Expressions of DLL4/Notch genes in RCC tissue samples or cell lines. (A and B) real－time PCR analyses of DLL4, Notch1, Notch2, Hes1, Hey1 and Hey2 mRNA expression in RCC samples (T; n = 120, 27, 27, 120, 120 and 120, respectively; ▲) relative to adjacent normal RCC samples (N; n = 10, 27, 27, 27, 19 and 20, respectively; △). mRNA levels were normalized to TATA box binding protein (TBP) and peptidylprolyl isomerase A (PPIA). (C) Western blot analysis showing protein levels of DLL4, Notch1, Notch2, Hes1, Hey1 and Hey2 in T relative to N. (D). DLL4 was not detected in all investigated RCC cell lines. Tumor samples were used as positive control, and β-actin was used as

reference. (E). Notch1 and Notch2 were both detected in all investigated RCC cell lines, and β-actin was used as reference.

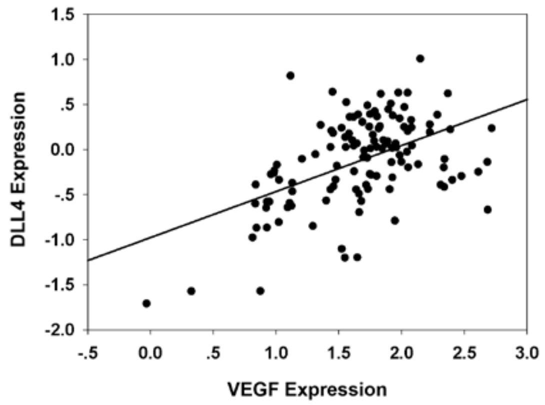

Figure S3. DLL4 expression positively correlates with VEGF expression (r = 0.499, p< 0.001, and n = 120).

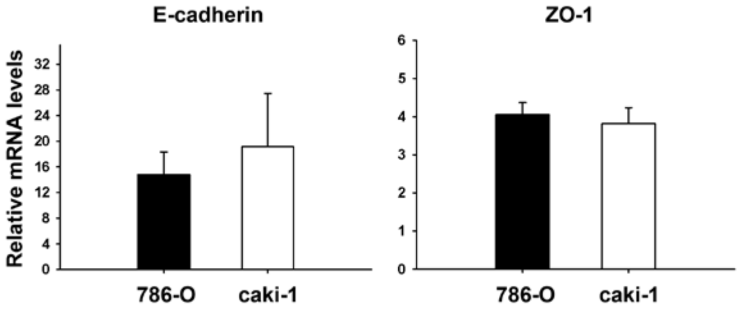

Figure S4: No significant difference of E–cadherin and ZO–1 was observed between 786-O and caki–1. Data were from three independent experiments and represented averages ± SEM.

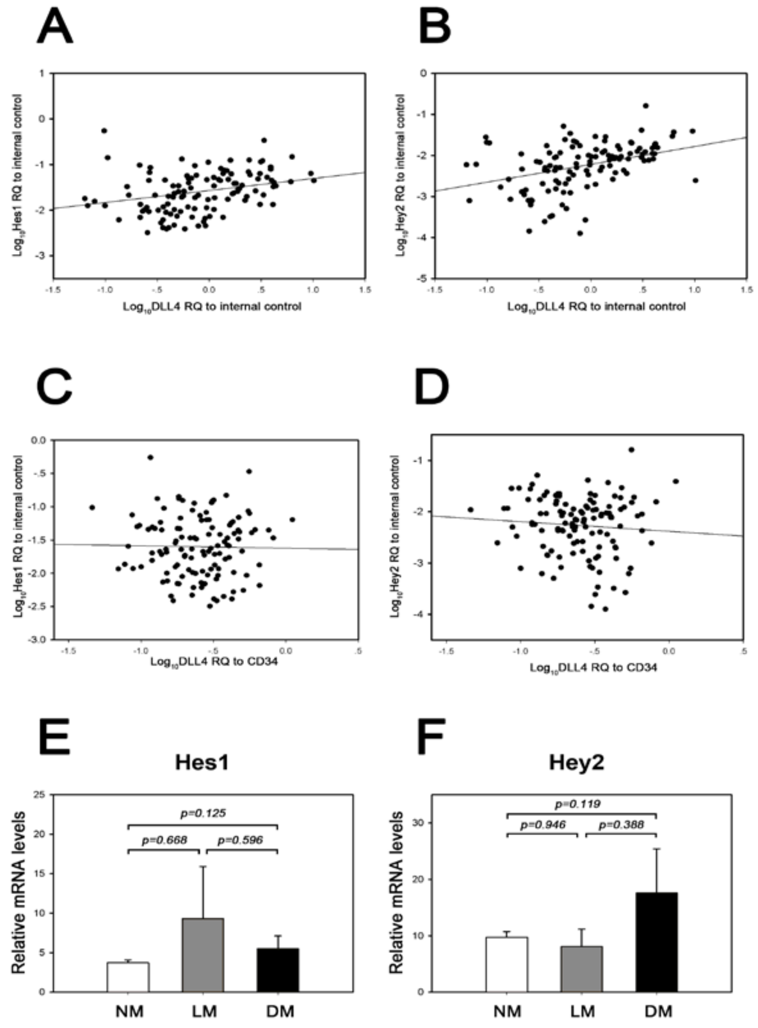

Figure S5. (A) Hes1 expression positively correlates with total DLL4 expression (r = 0.307, p= 0.001, and n = 120). (B) Hey2 expression positively correlates with total DLL4 expression (r = 0.397, p< 0.001, and n = 120). (C) Hes1 expression does not correlates with DLL4 density (n=120, p = 0.827). (D) Hey2 expression does not correlates with DLL4 density (n=120, p = 0.385). (E) No significant difference in Hes1 was observed among the NM, LM and DM groups. (F) No significant difference in Hey2 was observed among the NM, LM and DM groups. Data represent the means ± SEM.
